# The influence of shade allocation or total shade plus overhead fan on growth performance, efficiency of dietary energy utilization, and carcass characteristics of feedlot cattle under tropical ambient conditions

**DOI:** 10.5713/ajas.19.0112

**Published:** 2019-08-23

**Authors:** Beatriz I. Castro-Pérez, Alfredo Estrada-Angulo, Francisco G. Ríos-Rincón, Víctor H. Núñez-Benítez, Carlos R. Rivera-Méndez, Jesús D. Urías-Estrada, Richard A. Zinn, Alberto Barreras, Alejandro Plascencia

**Affiliations:** 1 Faculty of Veterinary Medicine and Zootechnics, Autonomous University of Sinaloa, Culiacán, Sinaloa 1084, México; 2 Sukarne Enterprise, Culiacán, Sinaloa 80300, México; 3 Department of Animal Science, University of California, Davis, CA 95616, USA; 4 Veterinary Science Research Institute, Autonomous University of Baja California, Mexicali, Baja California 21100, México

**Keywords:** Shade Allocation, Tropical Cattle, Feedlot Ration, Performance, Carcass

## Abstract

**Objective:**

The objective of this experiment was to evaluate the effect of shade allocation and shade plus fan on growth performance, dietary energy utilization and carcass characteristics of feedlot cattle under tropical ambient conditions

**Methods:**

Two trials were conducted, involving a total of 1,560 young bulls (289±22 kg BW) assigned to 24 pens (65 bulls/pen and 6 pens/treatment). Pens were 585 m^2^ with 15 m fence line feed bunks. Shade treatments (m^2^ shade/animal) were: i) limited shade (LS) to 1.2 m^2^ shade/animal (LS_1.2_); ii) limited shade to 2.4 m^2^ shade/animal (LS_2.4_); iii) total shade (TS) which correspond to 9 m^2^/animal, and iv) total shade equipped with fans (TS+F). Trials lasted 158 and 183 days. In both studies, the average weekly maximum temperature exceeded 34°C.

**Results:**

Increasing shade allocation tended (p = 0.08) to linearly increases average daily gain (ADG), and dry matter intake (DMI, quadratic effect, p = 0.03). This effect was most apparent between LS_1.2_ and LS_2.4_. Shade allocation, per se, did not affect gain efficiency or estimated dietary net energy (NE). Compared with TS, TS+F increased (p<0.05) ADG, gain efficiency, and tended (p = 0.06) to increase dietary NE. There was a quadratic effect of shade on *longissimus* area and marbling score, with values being lower (p<0.01) for LS_2.4_ than for LS_1.2_ or TS. Likewise, marbling score was lower for TS+F than for TS. Percentage kidney, pelvic, and heart (KPH) linearly decreased with increasing shade. In contrast, KPH was greater for TS than for TS+F.

**Conclusion:**

Providing more than 2.4 m^2^ shade/animal will not further enhance feedlot performance. The use of fans in combination with shade increases ADG and gain efficiency beyond that of shade, alone. These enhancements were not associated with increased DMI, but rather, to an amelioration of ambient temperature humidity index on maintenance energy requirement.

## INTRODUCTION

The importance of shade for enhancing feedlot cattle growth performance under conditions of elevated ambient temperature has been clearly demonstrated [1,2]. However, much remains to be understood regarding the nature and amount of shade required for optimal cattle performance, particularly under tropical conditions where feedlot cattle are exposed to high ambient temperatures coupled with high relative humidity (RH) and solar radiation for much of the year.

Umpapol et al [3] reported that provision of greater amounts of shade surface area to cattle finished in a subtropical environment markedly increased average daily gain (ADG) (10.4%) and dressing percentage (11.7%). Sullivan et al [4] conducted an experiment during the summer season in Queensland (subtropical climate) evaluating the impact of shade allowance (0, 2.0, 3.3, or 4.7 m^2^/animal) on growth performance in finishing cattle. They observed that the provision of greater than 2.0 m^2^ shade/animal did not further enhance growth performance, notwithstanding a high ambient high heat load. Preliminary studies [5] demonstrate that the combination of mechanical ventilation with conventional shading may provide further enhancements in cattle performance exposed to high heat loads in arid dry climates. Likewise, in tropical climates continuous ventilation facilitates growth performance negative effects of both elevated temperature and humidity [6]. Estrada-Angulo et al [7] observed that the net energy for maintenance (NE_m_) requirement of finishing cattle exposed elevated ambient temperatures under tropical conditions may increase by as much as 15%. Accordingly, we anticipate that the combination of ventilation and shade may minimize the combined negative effects of elevated ambient temperature and humidity on feedlot cattle performance. The objective of this experiment was to evaluate the influence of shade allocations or total shade plus overhead fan on growth performance, efficiency of dietary energy utilization, and carcass characteristics of feedlot cattle under tropical ambient conditions.

## MATERIALS AND METHODS

This experiment was conducted at a commercial feedlot (Empresas Sukarne) located near Culiacán, México (24° 46′ 13″ N and 107° 21′ 14″ W). Culiacán is about 55 m above sea level, and has a tropical climate. All animal management procedures were conducted within the guidelines approved by the Universidad Autónoma de Sinaloa Animal Use and Care Committee.

### Weather measurements and temperature humidity index estimation

Temperature (Ta) and RH were obtained every hour from an on-site weather station (Thermohygrometer Avaly; Mod. DTH880, Mofeg, Zapopan, Jalisco, Mexico) throughout the course of the study. The temperature humidity index (THI) was calculated using the following formula: THI = 0.81×Ta+ Hr (Ta−14.40)+46.40 [8].

### Animals management, diet and treatments

A total of 1,560 young bulls (approximately 50% zebu and the remainder represented by European breeds [Angus and Charolais in various proportions]; 289±22 kg) were used. Upon arrival bulls were vaccinated for IBR-PI_3_ (Express 5 HS, Boehringer Ingelheim Vetmedica, Inc., Duluth, GA, USA), clostridials-haemophilus (Caliber 7, Boehringer Ingelheim Vetmedica, Inc., USA), mannheimia hemolytic (One Shot, Zoetis, Florham Park, NJ, USA), treated for internal (Flukill, Virbac, Zapopan, JAL, México) and external parasites (Dectiver, Lapisa, La Piedad, MICH, México), injected with 500,000 IU of vitamin A (Compol AD3E, Lapisa, México), branded, ear-tagged, and implanted (Component TE 200, Elanco Animal Health, Inc., Greenfield, IN, USA). Cattle were re-implanted on d 80 of the trial. The study was conducted over a period of two years, involving two groups of 780 bulls each. The first trial was initiated on May 18, 2015 and harvested on October 23, 2015 (158 days on fed). The second trial was initiated on May 9, 2016 and harvested on November 11 2016 (183 days on fed). The same pens and treatments were used in both trials. Upon initiation of each trial, bulls were individually weighed, sorted by weight and randomly assigned within weight groupings to 12 pens (3 pens per treatment and 65 bulls/pen). Pens were 585 m^2^ (20.5 m length×28.5 m width), with 15 m north facing fence-line feed bunks. Four shade treatments were evaluated: i) limited shade (LS) to 1.2 m^2^ shade/animal (LS_1.2_); ii) limited shade to 2.4 m^2^ shade/animal (LS_2.4_); iii) total shade (TS) which correspond to 9 m^2^/animal), and iv) total shade equipped with fans operating continuously 24-h (TS+F). For LS treatments, shade material consisted of galvanized metal sheets (8.0 m length× 0.80 m width) secured side by side, widthwise, at a height of 3.5 m above the pen floor and oriented (east to west) parallel to the feed bunk. In the case of TS treatments, the entire pen surface was covered with high density polyethylene canvas (dome type structure, Empresas Invergrow, Culiacán, México) at a height of 6.3 m above the pen floor. While in TS+F treatment, fans (3 fans/pen) were aligned across the middle of the pen with one at the center and the other two located 4.3 m from the perimeter of the central fan. Fans (6 m diameter, equipped with 8 blades) were oriented horizontally at a height of 4.6 m above the pen floor, and were operating continuously 24-h. Each fan was powered by a 1,118.5 w (equivalent to 1.5 horsepower) motor, with a stated air displacement of 6023 cubic meters per minute (equivalent to 212,688 cubic feet per minute) and coverage area of 1,365 m^2^ (Bigvento Mod. BV06XA1508; Megaventilación, S.A., Guadalajara, Jalisco, México). Ingredient and nutrient composition of adaptation, preliminary and data collection diets are shown in Table 1. Steam flaked grains (corn and wheat) were prepared as follows. A chest situated directly above the rollers (46×61 cm corrugated) was filled to capacity (441 kg) with grain and then brought to a constant temperature at atmospheric pressure of 102°C using steam. The grain was steamed for approximately 20 min before starting the rollers. The first approximately 441 kg of steam-flaked grain was allowed to pass from the rollers before material was collected for use in the trial. This preliminary period served for warming the rolls and for adjusting the tension on the rolls to provide a flake density of 0.31 kg/L. Density measures were determined using a grain density scale (Weight Per Bushel Tester, Mill & Elevator Supply Co., Kansas City, MO, USA) on freshly processed grain obtained as it exited directly beneath the rolls. Steam-flaked grains were then spread on a concrete pad and allowed to air-dry for 3 days before feeding. Forages (alfalfa and corn stover) were ground in a hammer mill (Bear Cat #1A-S, Westerns Land and Roller Co., Hastings, NE, USA) with a 2.6-cm screen before incorporation into complete-mixed diets Additives, urea, limestone, and trace mineral salt were mixed in a 500 kg capacity horizontal mixer (SWLJ mixer, Shangai, China) for 5 min before being mixed with the remainder of ingredients in the basal diet. The total mixed ration was prepared in a horizontal mixer (Roto-Mix 720-18 cap 9,000 kg; Dodge City, KS, USA). Following by 7 to 8 min mixing period the feed was delivered to respective pens by feed truck (Roto-Mix mod. 540-14). Zilpaterol (6.4 mg/kg dry matter [DM]; Zilmax, MSD, Salud Animal, México) was included in the diet for 30 d followed by a 3-d withdrawal period before harvest. Cattle had *ad libitum* access to feed (offered in equal proportion at 0600 and 1300 h) and water. Samples of feed and orts were collected daily for DM analysis [9].

### Calculations

For calculation of growth performance, initial live weight (LW) is the off-truck arrival weight. Final LW was reduced 4% to adjust for digestive tract fill. Final shrunk weight was adjusted for HCW by dividing individual hot carcass weight (HCW) by the calculated average dressing percentage (HCW/0.6312). The ADG was computed by subtracting the initial weight from the final adjusted weight and dividing the result by the number of days on feed. Gain to feed ratio (gain efficiency) was determined by dividing ADG by the daily dry matter intake (DMI). The observed net energy (NE) content of the diet for maintenance and gain were calculated assuming a constant maintenance energy (EM, Mcal/d) cost of 0.077W^0.75^ and the required energy gain (EG) according to the equation: EG = (0.0493 W^0.75^) ADG^1.097^ [10]. Thus, the NE values of the diets for maintenance and gain were obtained by means of the quadratic formula:

$${\text{NE}}_{\text{m}}\;(\text{Mcal}/\text{kg})=(-b-\sqrt{b^{2}-4ac})/2\text{c}$$

[11],

where, a = −0.877DMI, b = 0.877EM+0.41DMI+EG, c = −0.41EM, and NE_g_ = 0.877NE_m_−0.41.

### Carcass data

Cattle were sacrificed in federally certified slaughterhouse located adjacent to the feedlot. Hot carcass weights were obtained at time of harvest. After carcasses chilled for 48 h, the following measurements were obtained: i) longissimus muscle (LM) area, taken by direct grid reading of the muscle at the 12th rib; ii) subcutaneous fat over the LM muscle at the 12th rib, taken at a location 3/4 the lateral length from the chine bone end (adjusted by eye for unusual fat distribution); iii) kidney, pelvic, and heart fat (KPH) as a percentage of HCW; and iv) marbling score using 2.0 as traces, 3.0 as minimum slight, 4.0 as minimum small, etc. [12].

### Pens surface condition

Beginning on week 15 of the study (day 98), pen surface conditions were visually evaluated four days weekly at 0800, 1150, and 1500 h during 9 consecutive weeks. Pen surfaces were classified as dry (no visual mud), slight mud (mud depth up to 5 cm), moderated mud (mud depth between 5 and 10 cm), and severe mud (mud depth greater than 10 cm).

### Statistical analyses

Feedlot performance data was analyzed as a generalized randomized block design, with block as trial replication and pen are the experimental unit. However, as the trial (block) by treatment interaction term was not significant, the F-test for treatments was based on the weighted average of the interaction and error term of the model, increasing the degrees of freedom for the error term and increasing the sensitivity of the F-test for treatment effects. Days on feed, as related to the dependent variables, was included as covariate in the additive model Y_ijk_ = μ+θ_i_+τ_j_+ɛ_ijk_. Where the covariate was statistically significant, least squares means were utilized in comparisons of treatment effect. Tukey pairwise comparisons of means was used. The MIXED procedure of SAS [13] was utilized to analyze the data. Carcass data were analyzed as a generalized complete block design with subsampling [14] with pen as the experimental unit and animal as the observational unit. The linear and quadratic contrast for shade allocation (1.2, 2.4, and 9 m^2^) treatment effects were tested by means of polynomial contrast constructed with “ORTHO” function in SAS for unequal spacing. Additionally, comparison between TS and TSF were contrasted using least significance difference. Pen floor conditions were analyzed using linear mixed model for repeated measures in a completely randomized design according to SAS [13] with covariance structure: unstructured (TYPE = UN), autoregressive [TYPE = AR(1)], and compound symmetric (TYPE = CS), and pen as a random component. Contrasts were considered significant when the p-value was ≤0.05, and tendencies were identified when the p-value was >0.05 and ≤0.10.

## RESULTS AND DISCUSSION

Average weekly climatic conditions during the course of the study are shown in Table 2. Minimum and maximum Ta averaged 18.6°C and 37.0°C, respectively. Average weekly maximum Ta exceeded 34°C for every week of the study. Relative humidity was 72.4%±7.7%. Average precipitation was lower for the first trial period (5.8 mm) than for the second trial period (26.9 mm), largely due to heavy precipitation during weeks 11 through 13 of second year. The minimum and maximum weekly THI averaged 62.5 and 97.4. Average daily THI was 80.9±2.1. Igono et al [15] proposed that the THI can be used to evaluate the environmental thermal stress. This index combines relative humidity and ambient temperature into a single value intended to reflect environmental conditions that influence the cattle’s ability to dissipate heat load. In accordance with nominal coding as: Normal THI <74; alert 75<THI<78; danger 79<THI<83; and emergency THI>84 [16], cattle experienced “danger” or “emergency” ambient conditions throughout the course of the study. Wind speed was low for both trials, averaging 4.5±0.7 and 4.5±0.5 km/h for 2015 and 2016, respectively. In both trials the predominant wind direction was north-northeast.

Treatment effects on pen surface conditions are shown in Table 3. Shade allocations, per se, did not affect (p>0.20) the proportion of total pen surface classified as “dry” or “slightly muddy”. The combination of fan and total shade (TSF) increased the proportion of pen surfaces that was dry, and decreased (p<0.05) the proportion of pen surface with moderate or severe mud. Although pen surfaces were dryer, no dust issues were noted with fan use. Compared with LS_1.2_ and LS_2.4_, TS markedly increased (p<0.05) the proportion of pen surface with moderate mud, but decreased (p<0.05) the proportion with severe mud. Pen surface conditions were similar for LS_1.2_ and LS_2.4_. As expected, the majority of severe mud accumulation was associated with cattle gathering directly beneath the shades. In open-pen conditions and absence of shade, severe mud negatively affects growth-performance [17] and dressing percentage of feedlot cattle [18]. However, under feedlot conditions where the more severe mud accumulation is largely occurring directly beneath shade structures, the direct impact of mud, per se, on growth-performance is less certain.

Treatment effects on growth performance and dietary energetics are shown in Table 4. Increasing shade allocation tended (linear effect, p = 0.08) to increase ADG and increased (quadratic effect, p = 0.03) DMI. Since all treatments received the same diets (equal dietary energy concentration, Table 1), changes in DMI directly reflect changes in energy intake. This effect was most apparent between LS_1.2_ and LS_2.4_. Shade allocation, per se, did not affect gain efficiency or estimated dietary NE. Thus, increasing shade allocation in the present experiment did not appear to affect the efficiency of energy utilization (partial efficiency of metabolizable energy for maintenance and gain) of cattle exposed to elevated THI. Instead, the improvement in ADG was due to increased energy intake. As well as exacerbating the effects of intake on dietary heat increment during periods of extreme ambient conditions, erratic patterns of intake have been associated with digestive disorders (day-to-day occurrence of subclinical acidosis) [19]. In the present study, variation in DMI (data not shown) was 27% lower in TSF group (coefficient of variation = 5.75% vs 7.89%) than in the LS_1.2_ group. Likewise, Barajas et al [2] observed that providing shade (3.3 m^2^/animal) to Brahman cross feedlot steers during a period of elevated temperature (average daily THI, 77) enhanced DMI, and hence ADG, but did not affect the observed vs expected dietary NE. This is consistent with the observation that cattle adapt to elevated (>75) THI by reducing DMI and associated metabolic heat load [8,20]. Nevertheless, with provision of shade, alone, the observed dietary NE averaged 94.5% of expected (Table 4). An alternative approach for expressing treatments effects on animal energetics in the present experiment is to keep the NE value of the diet constant and present treatment effects solely as a function of changes in the maintenance coefficient (MQ), as follows:

$$\text{MQ}=({\text{NE}}_{\text{m}}\times [\text{DMI}-(\text{EG}/{\text{NE}}_{\text{g}})])/{\text{SBW}}^{0.75}$$

where NE_m_ correspond to the NE_m_ of diet (Table 1), EG = (0.0493W^0.75^)ADG^1.097^ and SBW is the average shrunk body weight. Accordingly, elevated ambient THI for the treatments of shade without fans increased the MQ by 15.5% (MQ of 0.089 vs 0.077).

The objective of shade is to reduce cattle exposure to radiant energy. Under conditions of otherwise elevated THI (>75), the enhancement of feedlot cattle growth performance due to shade has been clearly demonstrated [2,4,21,22]. However, very little research has been reported that evaluates the amount of shade required to optimize this effect. It is generally considered that the provision of 2 m^2^/animal of shade is sufficient to optimize feedlot performance [23]. The basis for this recommendation is uncertain. Anecdotally, feedlot shades were typically constructed using standard 6.1 m corrugated galvanized steel panels hung in a line running parallel to the feed bunk. Considering the convention of 30.5 cm bunk space per head, this manner of construction provided 2 m^2^/animal of shade. Results of the present study are supportive of this recommendation.

Compared with TS, TSF increased ADG (10%, p<0.01), gain efficiency (8%, p = 0.02), and tended (p = 0.06) to increase dietary NE_m_ and NE_g_ (5% and 6%, respectively), with the ratio of observed-to-expected NE approaching 1. Thus, under the climatic conditions of this study, the influence of combining shade with overhead fans on dietary NE can be explained as a 15.5% reduction in maintenance energy expenditure. Likewise, Correa et al [5,24] observed that compared to shade alone (4 m^2^/animal), equipping shades with a series of fans to increase air flow increased ADG of feedlot cattle under conditions of elevated ambient temperature (THI>75).

As stated previously, whereas shade provides some degree of reduction in added effects of radiant energy exposure. It does not alleviate the combined impact of ambient temperature and relative humidity. When the ambient temperature exceeds 36°C, heat loss is largely via sweating and panting that is facilitated as cattle reach upper critical core body temperature. Air movement is an impact factor affecting both convective and evaporative heat losses [25,26]. For example, under condition of total shade, and average ambient temperature and relative humidity of 29°C and 72%, respectively (Table 2), increasing air speed from 0.2 to 2.5 m/s reduced the estimated heat load index [27] by 10%.

Treatment effects on carcass characteristics were inconsistent (Table 5). Provision of shade, per se, did not affect (p> 0.20) dressing percentage. However, dressing percentage was greater (1.8%, p<0.01) for TSF than for TS. This effect is likely the result of treatment effects on feeding patterns of cattle relative to time of harvest [28]. There were no treatment effects on fat thickness (p>0.20). Surprisingly, there was a quadratic effect of shade on LM area and marbling score, with values being lower (p<0.01) for LS_2.4_ than for LS_1.2_ or TS. Likewise, marbling score was lower (p = 0.01) for TSF than for TS. However, across treatments marbling scores were low, indicating only “traces” of visual marbling. Percentage KPH decreased (linear effect, p<0.01) with increasing shade. In contrast, KPH was greater for TS than TSF. The basis for these effects is not certain. Mitlöhner et al [21] observed that whereas provision of shade enhanced ADG, it did not affect carcass measures.

It is concluded that under the tropical ambient conditions of this study providing more than 2.4 m^2^ shade/animal will not further enhance feedlot performance. The use of fans in combination with shade increases ADG and gain efficiency beyond that of shade, alone. These enhancements were not associated with increased DMI, but rather, to an amelioration of ambient THI on maintenance energy requirement.

## Figures and Tables

**Table 1:** Composition of diets fed during the course of the study

Items	Diets fed1)		

	Adaptation	Preliminary	Finishing2)
Ingredient composition (%, DM)			
Steam-flaked corn	28.64	46.70	42.83
Steam-rolled wheat	-	-	26.81
Alfalfa hay	20.59	9.72	-
Corn stover3)	24.40	21.81	12.29
Molasses cane	13.40	11.96	9.64
Soybean meal	9.90	4.78	1.93
Tallow	1.51	2.87	3.50
Urea	0.50	0.80	1.20
Limestone	0.50	0.80	1.30
Trace mineral salt4)	0.56	0.56	0.50
Nutrient composition (DM basis)5)			
Net energy (Mcal/kg)			
Maintenance	1.63	1.87	2.14
Gain	1.02	1.23	1.43
Crude protein (%)	14.42	12.40	12.54
Calcium (%)	0.81	0.76	0.76
Phosphorus (%)	0.24	0.23	0.25

DM, dry matter.

1) Receiving diet was fed from d 1 to d 10, the transition diet from d 10 to d 18, and the finishing diet from d 18 until harvest.

2) Zilpaterol hydrochloride (ZIL; Zilmax, MSD, Salud Animal, México) was included in the diet according to label instructions at an inclusion rate of 6.4 mg/kg DM for 30 d followed by a 3 days withdrawal period before harvest.

3) Contained 6.2% crude protein, 74.4% neutral detergent fiber, and 43.6% acid detergent fiber.

4) Trace mineral salt contained salt 75%, microminerals 25% and was fortified with virginiamycin and monensin.

5) Nutrient composition and net energy values are based diet formulation and tabular values for individual feed ingredients [29].

**Table 2:** Ambient temperature, mean relative humidity, and mean temperature-humidity index

Week	Min T_a_ (°C)	Mean T_a_ (°C)	Max T_a_ (°C)	Min RH (%)	Mean RH (%)	Max RH (%)	Min THI1)	Mean THI	Max THI	Precipitation (mm)
1	19.71	27.29	35.49	33.23	60.22	85.77	64.13	76.27	93.24	0.30
2	18.59	27.18	36.35	23.61	52.82	79.94	62.45	75.17	93.39	2.60
3	23.00	29.30	36.52	36.43	62.92	87.60	68.16	79.51	95.36	10.50
4	24.73	29.29	35.12	46.08	67.50	85.57	71.19	80.18	92.58	8.60
5	24.61	30.22	36.75	40.60	63.91	83.40	70.48	80.99	94.81	3.60
6	25.76	30.62	36.74	45.44	67.05	86.05	72.43	82.08	95.38	17.10
7	25.61	30.39	36.79	45.21	67.77	87.94	72.21	81.85	95.89	64.40
8	25.31	30.48	36.69	45.71	68.85	91.04	71.89	82.16	96.41	78.20
9	25.16	30.34	36.29	47.08	70.30	90.44	71.85	82.18	95.59	82.00
10	24.77	29.55	35.72	52.94	75.26	93.89	71.95	81.74	95.35	33.40
11	24.71	29.63	35.99	54.14	78.70	95.24	72.00	82.39	96.11	176.50
12	25.99	30.48	36.99	50.42	74.52	92.94	73.30	83.07	97.36	90.30
13	25.56	29.91	35.91	52.45	75.73	93.52	72.96	82.37	95.60	56.30
14	24.58	28.65	34.01	59.65	83.19	97.84	72.38	81.46	93.05	9.70
15	24.84	29.17	35.18	55.83	80.06	95.89	72.35	81.85	94.82	29.40
16	25.46	29.66	35.83	55.43	80.67	96.63	73.15	82.73	96.13	10.50
17	24.77	29.05	34.24	59.44	81.84	97.79	72.63	81.92	93.54	0.00
18	25.79	29.74	35.24	56.59	80.01	95.47	73.74	82.76	94.84	0.00
19	25.54	29.22	34.79	60.37	82.54	96.08	73.81	82.30	94.17	0.00
20	25.27	29.62	36.14	51.13	77.49	94.03	72.43	82.19	96.12	0.00
21	23.25	27.95	34.40	49.56	75.20	92.91	69.62	79.23	92.85	0.00
22	24.91	29.94	36.69	49.91	74.45	90.84	71.82	82.22	96.37	0.20
23	23.62	29.26	37.04	38.79	67.13	87.98	69.11	80.08	96.32	0.20
24	22.87	28.33	35.75	39.21	68.14	88.82	68.25	78.84	94.32	0.20
25	20.93	26.55	34.25	42.12	72.60	92.87	66.10	76.73	92.58	0.00
Avg	24.21	29.27	35.79	47.65	72.35	91.22	70.81	80.89	94.88	26.96
SD	1.91	1.09	0.93	8.97	7.70	4.75	2.86	2.11	1.40	42.80

T_a_, ambient temperature; RH, relative humidity; THI, temperature-humidity index; SD, standard deviation.

1) THI = 0.81×ambient temperature+[(relative humidity/100)×(ambient temperature−14.4)]+46.40 [8].

**Table 3:** Influence of shade treatments on pen surface conditions1)

Items	Shade treatments2)				SEM

	LS_1.2_	LS_2.4_	TS	TSF	
Weeks of evaluation	9	9	9	9	-
Pens	6	6	6	6	-
Pen floor, as % of surface3)					
Dry	25.37^a^	22.76^a^	13.03^a^	67.53^b^	9.75
Light mud	21.80	21.35	43.28	28.78	13.07
Moderate mud	11.85^a^	7.38^a^	41.29^b^	2.77^c^	7.43
Severe mud	40.98^a^	48.51^a^	2.40^b^	0.92^b^	10.68

SEM, standard error of the mean; THI, temperature-humidity index.

1) Observations made at 08:30, 11:30, and 15:30 from week 15 to week 23 of the study (average THI: Min = 72.07±1.67; Mean = 81.69±1.21; Max = 95.02±1.31).

2) LS_1.2_, low allocation than recommended (1.2 m^2^/shade/animal); LS_2.4_, 2.4 m^2^/shade/animal; TS, totally shaded (9 m^2^/shade/animal); TSF, totally shaded (9 m^2^/shade/animal) plus fan.

3) Pen surface conditions were visually evaluated three hours daily (0:800, 11:50, and 15:00 h), 4 days a week during consecutive 9 weeks. Pen surfaces were classified as dry (no visual mud), slight mud (mud depth up to 5 cm), moderated mud (mud depth between 5 and 10 cm, and severe mud (mud depth greater than 10 cm [17].

a–c Means in a row with different superscripts differ (p<0.05).

**Table 4:** Influence of shade treatments on growth performance and dietary energy of feedlot steers

Items	Shade treatments1)				SEM	p-value		
	
	LS_1.2_	LS_2.4_	TS	TSF		Linear	Quadratic	TS vs TSF
Days on fed	172	172	172	172	-	-	-	-
Pens	6	6	6	6	-	-	-	-
Weight (kg)								
Initial	286.5	286.8	287.1	287.2	1.2	0.74	0.89	0.97
Final	482.9^a^	490.7^a^	492.8^a^	512.4^b^	4.9	0.12	0.19	<0.01
Weight gain (kg/d)	1.15^a^	1.19^a^	1.20^a^	1.32^b^	0.02	0.08	0.26	<0.01
DM intake (kg/d)	7.06^a^	7.59^b^	7.49^b^	7.62^b^	0.14	0.21	0.03	0.53
Net energy intake (Mcal/d)								
Maintenance	15.11^a^	16.24^b^	16.02^b^	16.30^b^	0.29	0.21	0.03	0.53
Gain	10.10^a^	10.85^b^	10.71^b^	10.90^b^	0.20	0.21	0.03	0.53
Gain to feed	0.163^ab^	0.158^a^	0.162^ab^	0.175^b^	0.004	0.90	0.33	0.02
Observed NE (Mcal/kg)								
Maintenance	2.00^ab^	1.93^a^	1.97^ab^	2.07^b^	0.03	0.89	0.18	0.06
Gain	1.34^ab^	1.28^a^	1.32^ab^	1.40^b^	0.03	0.89	0.18	0.06
NE (observed-to-expected)								
Maintenance	0.960^ab^	0.928^a^	0.946^ab^	0.992^b^	0.016	0.89	0.18	0.06
Gain	0.949^ab^	0.907^a^	0.930^ab^	0.990^b^	0.021	0.89	0.18	0.06
Observed to expected DMI	1.048^ab^	1.093^a^	1.073^ab^	1.010^b^	0.015	0.75	0.15	0.05

SEM, standard error of the mean; NE, net energy; DMI, dry matter intake.

1) LS_1.2_, low allocation than recommended (1.2 m^2^/shade/animal); LS_2.4_, 2.4 m^2^/shade/animal; TS, totally shaded (9 m^2^/shade/animal); TSF, totally shaded (9 m^2^/shade/animal) plus fan.

a,b Means in a row with different superscripts differ (p<0.05).

**Table 5:** Influence of shade treatments on carcass characteristics of feedlot steers

Items	Shade treatments1)				SEM	p-value		
	
	LS_1.2_	LS_2.4_	TS	TSF		Linear	Quadratic	TS vs TSF
HCW	305.0^a^	309.4^a^	310.8^a^	322.9^b^	3.2	0.31	0.36	<0.01
Dressing percentage	63.0^a^	63.1^a^	62.8^a^	63.9^b^	0.2	0.28	0.63	<0.01
LM area (cm^2^)	89.9^a^	86.4^b^	89.5^a^	89.2^a^	0.9	0.39	<0.01	0.81
Fat thickness (cm)	0.60	0.55	0.55	0.58	0.25	0.35	0.23	0.37
KPH (%)	1.80^a^	1.59^bc^	1.49^b^	1.63^c^	0.037	<0.01	0.11	0.02
Marbling score2)	3.14^a^	2.76^b^	2.98^c^	2.71^b^	0.06	0.79	<0.01	0.01

SEM, standard error of the mean; HCW, hot carcass weight; LM, longissimus muscle; KPH, kidney, pelvic, and heart.

1) LS_1.2_, low allocation than recommended (1.2 m^2^/shade/animal); LS_2.4_, 2.4 m^2^/shade/animal; TS, totally shaded (9 m^2^/shade/animal); TSF, totally shaded (9 m^2^/shade/animal) plus fan.

2) Coded as USDA [12]: Traces, 2.0; minimum slight, 3.0; minimum small, 4.0, etc.

a–c Means in a row with different superscripts differ (p<0.05).
